# Association of CT Scan Parameters with the Risk of Renal Angiomyolipoma Rupture; a Brief Report

**DOI:** 10.22037/aaem.v10i1.1472

**Published:** 2022-01-01

**Authors:** Razieh Heidari, Mostafa Ghadamzadeh, Mansour Bahardoust, Forugh Khezrian, Afrooz Moradkhani, Parmida Ghadimi, Seyed Morteza Bagheri

**Affiliations:** 1 Department of Radiology, Iran University of Medical Sciences, Tehran, Iran.; 2 Department of Radiology, Hasheminejad Kidney Center (HKC), Iran University of Medical Sciences, Tehran, Iran.; 3 Department of Epidemiology, School of Public Health, Shahid Beheshti University of Medical Sciences, Tehran, Iran.; 4 Factually of Medicine, Iran University of Medical Sciences, Tehran, Iran.

**Keywords:** Angiomyolipoma, kidney, tomography, X-ray computed, neoplasms, rupture, emergencies

## Abstract

**Introduction:**

Rupture of renal angiomyolipoma (AML) is an emergency and life-threatening complication. This study aimed to evaluate the association of computed tomography (CT) scan parameters with the risk of rupture in renal AMLs.

**Methods:**

In this retrospective cross-sectional study, patients who were referred to a referral university hospital with diagnosis of AML, between 2007 and 2019, were included. Patients were divided into ruptured and non-ruptured cases based on surgery and CT scan findings and the baseline characteristics as well as CT scan parameters were compared between the two groups.

**Results:**

20 AML patients with the mean age of 39.6 ± 12.5 years were included (75% female). The lesion was ruptured in 8 (40%) patients. The mean size of the lesion was ‎97.0 ± 15.9 mm‎‏ in the ruptured and ‏‎72.0 ± 29.4‎‏ in the non‎-‎ruptured AML ‏‎(‎p ‎= ‎‏0.045). The ‎mean fat density based on non-contrast enhanced CT (NCCT) scan (-‎56.1 ± 16.3 ‎vs ‎-‎‎74.9±24.1; ‏p = ‏‎0.018) and contrast enhanced CT (CECT) scan (-‎20.8 ± 16.9 ‎vs ‎-‎‎50.5 ± 31.7; ‏p ‏= ‏‎0.‎016) was significantly higher in the ruptured cases. Total tumor density based on NCCT scan was significantly greater in the ruptured ‎AMLs ‏(‏‎19.6 ± 25.9 ‎‏ vs‎ ‎-22.7±41.6, p=0.033).

**Conclusion:**

It seems that some CT scan parameters such as mean fat density and ‎total tumor density ‏could be used for differentiation between ruptured and non-ruptured AMLs.

## 1. Introduction

Angiomyolipoma (AML) is the most common benign tumor of the kidney, composed of blood vessels, muscle, and fat, with a prevalence of 0.2 - 0.6% and a strong female predilection (1). They are asymptomatic in most patients, and the diagnosis is usually made incidentally. The lesion remains stable in many patients and is managed with periodic evaluations and close monitoring of the lesion’s behavior. However, it could become complicated with tumor rupture and retroperitoneal hemorrhage, which are considered emergency and life-threatening consequences (2).

To avoid the life-threatening consequences of AML hemorrhage, the risk of tumor rupture is determined based on the tumor size, and surgical intervention is generally performed for tumors sized 4 to 8 cm (3, 4). However, a smaller size does not always preclude the risk of tumor rupture, and AML hemorrhage has also been reported in tumors smaller than 4cm (5). In addition, several other risk factors have also been reported to be associated with AML rupture in recent studies (5). Many precipitating factors trigger AML rupture, pregnancy plays an important role in tumor growth and rupture, genetic alteration is also the innate reason for tumor rupture (5). Therefore, further characterization of patients at high risk for rupture is necessary to prevent under-treatment of the patients.

Imaging, particularly computed tomography (CT) scan, plays a critical role in diagnosing and managing renal AMLs. In addition to mass size, the identification of adipose tissue is the cornerstone of the diagnosis of the classic AML (2). Limited studies have investigated the risk factors associated with rupture and their prognosis (6). This study aimed to evaluate the association of computed tomography (CT) scan parameters with the risk of rupture in renal AMLs.

## 2. Methods

***2.1 Study design and setting***

In this retrospective cross-sectional study, patients who were referred to a referral university hospital with an imaging diagnosis of AML (lipomatous components in the tumor mass observed on Non-enhanced CT scan), between 2007 and 2019, were included. Patients were divided into ruptured and non-ruptured cases based on surgery report in patients with available data and CT findings including mass irregularity, heterogenicity, and free fluid in abdominopelvic cavity. The baseline characteristics as well as CT scan parameters were compared between the two groups. 

This study was approved in Ethics Committee of Iran University of Medical Sciences (ethical code: IR.IUMS.REC. 1399.636), Tehran, Iran. The research team of this study adhered to the ethical principles of the Helsinki Convention regarding clinical studies.

***2.2 Participants***

Sampling was done consecutively, choosing the cases available during the study period. Patients with a prior treatment history and inadequate imaging records were excluded from the study. 

***2.3 Data gathering***

The demographic characteristics of the patients, including age and gender were extracted from the patients' medical profiles. In addition, patients’ CT scan records were reviewed for the laterality of the lesion(s), number of the lesions, tumor size, vessel diameter, fat percentage, and total and subtotal fat density based on non-contrast enhanced CT (NCCT) scan and contrast enhanced CT (CECT) scan, as well as total contrast enhancement. 

The largest tumor diameter was regarded as the tumor size. The fat percentage was reported based on the visual estimate of the fat content detectable by CT scan (7). Accordingly, the AMLs were categorized into three subsets including fat-low (≤25%), fat-moderate (25-75%), and fat-rich (≥75) lesions. Two expert radiologists separately estimated the fat content. In case of any disagreement between the two observers, a third radiologist was consulted to reach a consensus.

***2.4 Statistical analysis***

SPSS for Windows, version 16 (SPSS Inc., Chicago, Ill., USA), was used for the statistical analysis of data. Descriptive statistics were provided as mean ± standard deviation or number and percentage. A comparison of the mean between the two groups was made using a Mann–Whitney U test. Categorical variables were compared using the chi-square or fisher’s exact test. A P-value <0.05 was considered significant.

## 3. Results

***3. 1 Baseline characteristics of studied cases***

Finally, 20 AML patients with the mean age of 39.6±12.5 years were studied, in 8 (40%) of whom the lesion was ruptured (75% female). Table 1 compares the baseline characteristics of cases between ruptured and non-ruptured cases. The two groups were similar regarding the mean age (p = 0.9), gender (p = 0.7), number of lesions (p = 0.15), and laterality of lesion (p = 0.26). 

***3.2 CT scan characteristics***

In the ruptured group, the lesion was fat-low in 2 (25%) patients and fat-moderate in 6 (75%) patients. In the non-ruptured group, the lesion was fat-low in 3 (25%) patients, fat-moderate in 6 (50%) patients, and fat-rich in 3 (25%) patients. The fat percentage was not significantly different between the two groups (p = 0.29). Table 2 compares the CT scan characteristics between two groups. Fat density based on NCCT (p = 0.018), fat density based on CECT (p = 0.016), and total tumor density based on NCCT (p = 0.033) were significantly higher in ruptured cases. 

**Table 1:** Comparison of baseline characteristics between the ruptured and non-ruptured renal angiomyolipoma cases

**Variable**	**Ruptured ** **(n=8)**	**Non-ruptured ** **(n=12)**	**P-value**
Age(year)	40.2±10.3	39.3±14	0.9
**Gender**			
Male	2 (25.0)	3 (25.0)	0.7
Female	6 (75.0)	9 (75.0)	
**Number of lesions**			
1	4 (50.0)	6 (50.0)	0.15
2	2 (25.0)	0 (0.0)	
>2	2 (25.0)	6 (50.0)	
**Laterality**			
Left	2 (25.0)	6 (50.0)	0.26
Right	6 (75.0)	6 (50.0)	
**Pseudo-aneurysm**			
No	7 (87.5)	11 (91.7)	0.65
Yes	1 (12.5)	1 (8.3)	

Data are presented as mean ± standard deviation or number (%).

**Table 2:** Comparison of computed tomography scan characteristics between the ruptured and non-ruptured renal angiomyolipoma (AMLs)

**Variable**	**Ruptured** ** (n=8)**	**Non-ruptured** **(n=12)**	**P-value**
**Fat content**			
Low	2 (25.0)	3 (25.0)	0.29
Moderate	6 (75.0)	6 (50.0)	
Rich	0 (0.0)	3 (25.0)	
**Tumor size (mm** * **)**	‎97±15.9‎	‎72±29.4‎	0.045
**Vessel diameter (mm)**	‎4.2±2.3‎	‎3.8± 2‎	0.65
**Total density after contrast ** **‎** ** (** HU** **) ** **‎**	‎43.8±42.4 ‎	‎21.8±72.9‎	0.27
**Highest density after contrast** **‎** ** (** ***HU*** **) ** **‎**	‎85.6±36.5 ‎	‎78±41.5 ‎	0.49
**Soft-tissue density after contrast ** **‎** **(** ***HU*** **) ** **‎**	‎74.4±32.9 ‎	‎75.5±53.7 ‎	0.81
**Fat density before contrast ** **‎** **(g/cm** ^3***^ **)**	-56.1±16.3	-74.9±24.1	0.018
**Fat density after contrast ** **‎‎** **(** ***HU*** **) ** **‎**	‎-20.8±16.9 ‎	‎-50.5±31.7 ‎	0.016
**Total density without contrast ** **‎‎‎** **(** ***HU*** **) ** **‎**	‎19.6±25.9 ‎	‎-22.7±41.6 ‎	0.033
**Highest density without contrast ** **‎‎‎‎** **(** ***HU*** **) ** **‎**	‎44.5±28.6 ‎	‎38.7±50 ‎	0.37
**Total enhancement (HU)**	‎24.1±20.1 ‎	‎32.6±16.3‎	0.13
**Fat enhancement (HU)**	35.5±11.8	24.4±10.1	0.068

Data are presented as mean ± standard deviation or number (%). *mm: millimeter; **:HU: Hounsfield unit; ***: g/cm3: gram per cubic centimeter.

## 4. Discussion

In this study, we evaluated the role of CT scan parameters in predicting the rupture of renal AML. CT scan parameters, particularly fat ‎density based on NCCT and CECT ‎and ‏total tumor density based on NCCT, ‎were significantly higher in patients ‏with a ruptured AML. 

Tumor size was significantly larger in the ruptured AMLs. No significant ‏association was found between other CT parameters and the risk of AML rupture. No significant ‏association was found between the demographic characteristics of the patients and the risk of ‏AML rupture, as well. ‏

It is widely accepted that an AML size of >4cm is associated with a higher risk of tumor rupture. ‏In the present study, the size of AML was greater in the ruptured AMLs. However, the ‏size of the lesion was also more than 4cm in the majority of non-ruptured AML. These ‏observations suggest a predictive role for other AML rupture parameters besides the tumor size. ‏

Wang et al.‏‎ reviewed the recent radiological and clinical findings associated with AML rupture. ‎According to their results, in addition to the lesion size, genetic abnormality, aneurysm formation, and pregnancy were also ‎associated with the risk of AML rupture. They concluded that any ‎decision for surgical intervention in AML should be based on the cumulative effects of the ‎introduced risk factors and not just on the tumor size ‎(5).

Yamakado et al. evaluated the association of tumor size and aneurysm formation with spontaneous rupture in 29 kidneys with AML. Tumor and aneurysm sizes were compared between the ruptured and non-ruptured AML. The lesion was hemorrhagic in 8 patients. According to their results, tumor size was >4 cm and aneurysm size was ≥5 mm in all hemorrhagic lesions. Multiple regression analysis revealed that aneurysm size was the most important predictive factor for AML rupture. However, the size of an aneurysm seems to be corresponding to the tumor size, and larger aneurysms often appear in larger tumors. Therefore, the size of an aneurysm could be a reflection of tumor size (3). 

Gandhi et al. used 64 slices of multidetector computed tomography and angiography to investigate the association of tumor size and aneurysm formation with spontaneous rupture of renal AML in 27 patients (34 kidneys, 6 ruptures, and 28 non-ruptured AMLs) (8). In contrast to the study of Yamakado et al. (3), tumor and aneurysm size could not predict the spontaneous rupture of the AML in the study of Gandhi et al. These results indicate the need for further studies evaluating factors affecting the rupture of AML.

Several other studies have also attempted to establish novel risk factors for AML rupture (9, 10). However, the attempt to find more reliable markers of AML rupture continues. CT scan is an imperative imaging modality in the diagnosis of AML (11). Some authors have used CT finding to estimate the risk of rupture in AML (12, 13). Recent evidence suggests an association between fat content and rupture of AML (14). Accordingly, we hypothesized that quantifying the fat content of the AML based on CT images could ‎be used as a predictive factor of lesion rupture. According to our results, fat density based on NCCT and CECT ‎and total tumor density based on NCCT were‏ significantly higher in the ruptured AMLs. ‏Therefore, they could be promising parameters for predicting AML rupture and performing ‏prophylactic‏ surgery/intervention. 

## 5. Limitations

Despite the promising findings of this study, it should be noted that the present results might be flawed for ‏several reasons and further studies are required to confirm the results obtained here. Firstly, it ‏was a retrospective study with potential information bias. Secondly, the small number of ‏patients might have affected the power of the statistical analysis. Finally, the number of patients was ‏not large enough to perform multivariate analysis. ‏

## 6. Conclusions

CT scan parameters‎, ‎particularly ‏fat density based on NCCT and CECT ‎and total tumor density based on NCCT, could be ‎used for predicting the risk of rupture in renal AML and performing prophylactic‏ surgery/intervention to prevent ‏life-threatening consequences of the AML ‏hemorrhage‏. Despite the promising role of CT scan parameter in ‏this differentiation, the results need to be re-confirmed in prospective large-scale studies. ‏

## 7. Appendix

### 7.1. Acknowledgments

Hereby, the authors would like to express gratitude to the guidance and advice from the “Clinical Research Development Unit of Hasheminejad Kidney Center (HKC), Tehran, Iran”.

### 7.2 Author contribution

All the authors of this study met the standard criteria of authorship based on the recommendations of the International Committee of Medical Journal Editors.

### 7.3 Funding

 No funding was received for this study.

### 7.4 Conflict of interest

None declared.
